# Factors Associated with Depression, Anxiety, and Somatic Symptoms among International Salespeople in the Medical Device Industry: A Cross-Sectional Study in China

**DOI:** 10.3390/healthcare11152174

**Published:** 2023-07-31

**Authors:** Beibei Mao, Penkarn Kanjanarat, Tinakon Wongpakaran, Unchalee Permsuwan, Ronald O’Donnell

**Affiliations:** 1 Master of Science Program (Mental Health), Graduate School, Chiang Mai University, Chiang Mai 50200, Thailand; beibei_m@cmu.ac.th (B.M.); unchalee.permsuwan@gmail.com (U.P.); ronald.odonnell@asu.edu (R.O.); 2 Department of Pharmaceutical Care, Faculty of Pharmacy, Chiang Mai University, Chiang Mai 50200, Thailand; 3 Department of Psychiatry, Faculty of Medicine, Chiang Mai University, Chiang Mai 50200, Thailand; 4 Behavioral Health, College of Health Solutions, Arizona State University, Phoenix, AZ 85004, USA

**Keywords:** depression, anxiety, somatic symptom, organizational factor, occupational factor, effort–reward imbalance, health-promoting leadership, health climate, inner strength, perceived social support, international salespeople, medical device industry

## Abstract

Background: The physical and mental health of corporate employees is equally important, especially for international salespeople in the in vitro diagnostic (IVD) medical device industry. The rapid growth of the IVD market is driven by the increasing prevalence of chronic and infectious diseases. This study aims to determine the prevalence of depression, anxiety, and somatic symptoms among international salespeople in China’s IVD industry and identify the association of socio-demographic, occupational, organizational, and psychosocial factors with mental health outcomes for depression, anxiety, and somatic symptoms in Chinese IVD international salespeople. Methods: The study was a cross-sectional survey of international salespeople (ISs) in IVD companies officially registered in China. An online survey was designed to collect data through email contact with IVD companies and social media between August 2022 and March 2023. Measured factors included effort-reward imbalance (ERI), health-promoting leadership (HPL), health climate (HC), inner strength (IS), and perceived social support (PSS). Mental health outcomes assessed using the Core Symptom Index (CSI) were depression, anxiety, and somatic symptoms. Results: A total of 244 salespeople responded to the survey. CSI scores indicated that 18.4% (n = 45) and 10.2% (n = 25) of the respondents had symptoms of major depression and anxiety, respectively. ERI was positively correlated, while the IS and PSS were negatively correlated with major depression, anxiety, and somatic symptoms (*p* < 0.01). The health climate was negatively correlated with major depression (*p* < 0.05). Education background was associated with somatic symptoms (*p* < 0.05). ERI, IS, and gender were significant predictors of major depression, anxiety, and somatic symptoms (*p* < 0.05). Conclusion: The prevalence of depression and anxiety in China’s IVD international salespeople was considered low compared with the prevalence in Chinese populations during COVID-19 but higher than those before the pandemic. Effort–reward imbalance, inner strength, and gender were significant factors in major depression, anxiety, and somatic symptoms among IVD international salespeople.

## 1. Introduction

The physical and mental health of working-age adults around the world is facing unprecedented challenges. Studies revealed that 14.7% of people in the UK, 17.3% in the European Union, 20% in Australia, nearly 18.3% in the United States, and 33% in Latin America experience a mental health problem in the workplace [1,2,3,4,5]. In China, a survey by the State Council reported that 20% of employees felt stressed, and 87.4% of Chinese entrepreneurs experienced emotional symptoms such as stress, irritability, fatigue, depression, pessimism, and disappointment [6]. In 2019, among Chinese adults, the prevalence of anxiety disorders was 5.0%, and that of mood disorders was 4.1% [7]. Work-related stressors can also affect physical health, with reported high rates of chronic diseases such as hypertension, hyperlipidemia, and chronic gastritis among business operators [8]. In addition, the prevalence of mental disorders comes with enormous economic costs. Anxiety and depression reduce productivity and cost the global economy USD 1 trillion annually [9]. The current global crisis from COVID-19 [10,11], war and military conflicts, and climate change has caused economic decline and increased poverty [12]. Enterprise employees face increased risks of adverse mental health outcomes [13]. Understanding employee mental health and related influencing factors is an important issue to be explored, especially in high-growth markets such as the in vitro diagnostic (IVD) industry.

The IVD industry produces medical devices such as COVID-19 rapid test kits; driven by the coronavirus pandemic, the industry has made significant progress [14]. According to an overview of IVD market trends, the global IVD market increased by more than 54% in 2021 and reached USD 77 billion in 2022 [15]. The Kalorama market report for 2021, “Global IVD Market, 10th Edition”, showed that the compound growth rate of China’s IVD market from 2016 to 2021 ranked first among all countries, and it continues to grow significantly [16]. The development and application of IVD products have created more employment opportunities. The data show that, in 2014, the European IVD industry provided direct employment opportunities for 75,000 people [17]. IVD sales representatives serve as a link between healthcare professionals and medical device companies and are an indispensable part of the interface between IVDs and surgeons [18]. International salespeople account for between 5% and 20% of the labor force in the industry [19]. The results from a survey by the McKinsey Health Institute on the mental health of employees across 15 countries showed that approximately 60% of employees have experienced at least one mental health challenge at some point in their lives. The results showed that employees in healthcare systems and services, including the IVD industry, face mental health challenges as much as employees in other industries, including technology, travel transport and logistics, consumer and retail, asset management, global energy and materials, and professional, scientific, and technical services [20]. Salespeople in IVD industries are facing mental health risks at the workplace as well as in other fast-growing industries, such as excessive workload, long work hours, job insecurities, conflicts in home/work demands, and limited support from colleagues [13]. In addition, these employees face work-related risks such as long travel times to visit clients, long working hours to accommodate time differences, the high pressure of sales targets and performance, and during COVID-19 remote work [21,22]. Previous studies found that these occupational factors were associated with psychological problems such as obsessive-compulsive disorder, anxiety, depression, suicide, and somatization [21,23,24].

### 1.1. Job Obligations and Responsibilities of IVD International Salespeople

IVD international salespeople have numerous job obligations and responsibilities. First, IVD international salespeople must fully comprehend product information and adhere to all standard operating procedures. They must collaborate closely with medical agents to achieve sales targets. Furthermore, another job obligation of IVD international salespeople is to provide product knowledge training to the sales team and regional agents and assist in various marketing activities. Management and leadership skills and the ability to work independently and communicate across departments to complete bidding, registration, shipping, and after-sales are also required. Because of such high-demand obligations and responsibilities, it has been demonstrated that a lack of family time, feelings of inability to cope with work, and pressure to continually improve performance are associated with an increased risk of depression [21,22,25]. It has been reported that long-term sleep deprivation, frequent alcohol consumption, and lower blood pressure—all common factors among international business travelers—are associated with depression [25,26]. In addition, jet lag, a common problem related to traveling, disrupts the body’s circadian rhythm and has profound effects on cognitive function among international salespeople [25].

### 1.2. Effort–Reward Imbalance

The effort–reward imbalance (ERI) in the occupational environment is related to employee physical and mental health problems [27]. The imbalance of effort and reward is measured by the ratio of effort and reward, reflecting whether the individual is in a state of high effort and low reward [28]. The Asian IVD industry started late and developed rapidly, and the employee promotion mechanism and salary structure are immature. Research has verified that the job stress caused by ERI increases the risk of job burnout and depression in sales positions [29]. International salespeople in the IVD industry might have experienced an even tougher ERI situation during the COVID-19 outbreak. Whether ERI is an important predictor of depression, anxiety, and somatic symptoms in IVD international salespeople remains unknown.

### 1.3. Health-Promoting Leadership and Health Climate

Two organizational factors, health-promoting leadership (HPL) and health climate (HC), have been widely studied among employees across industries. The mismatch of HPL and HC between employees and organizations affects the health of the employees [30]. Leaders in an organization are a key factor in promoting employee wellness because of their ubiquitous impact on employees. The “Health-Promoting Leadership” model has attracted increasing attention in occupational health science [31]. HPL is a leadership style that can improve positive employee work behaviors and reduce stress; this style directly demonstrates health concepts or healthy working conditions to employees, reasonably arranges workload, and establishes a scientific salary system [32,33]. Related studies have confirmed that health-oriented leadership, health awareness, health value, and health behavior scores were significantly associated with individual employee depression and anxiety symptoms [34]. Along the same lines, organizational climate is found to be an important part of the work environment [35]. HC, the sum of health resources, health elements, and health-centered communication in an organization [36], impacts employees’ health [37]. For example, by offering healthy lunches, the employees developed healthy eating habits [38]. A healthy climate can influence employees to form a healthy life concept, and employees display more fitness behaviors and higher job satisfaction [33]. Research has revealed that in organizations providing greater support for employee health issues, employees experience less work stress [39].

### 1.4. Positive Mental Health

Positive mental health, such as inner strength and character strength, is evidently related to mental health well-being. In Theravada Buddhism, ten positive psychological characteristics are referred to as “perfections” [40] and are described as character strengths by Seligman and colleagues [41]. When these “perfections” are applied in psychotherapy, they are called “inner strengths” [42]. Character strengths such as perseverance and patience allow an individual to adapt well when faced with stressors. Related research supports the idea that inner strength produces a protective effect against depression and mediates the relationship between depression and self-rated health [43,44]. Inner strength, e.g., wisdom, perseverance, and determination [45], is a predictor of well-being and a buffering factor for mental health problems [46]. However, the effect of inner strength has not yet been tested on IVD international salespeople. Social support is another positive mental health factor found to reduce psychological distress in the face of stressful events [47]. There are many forms of social support, including support from family, friends, and significant others. Evidence shows that support from supervisors is an effective source of social support for reducing employee burnout [48]. Overall social support is negatively correlated with depressive symptoms [49,50].

### 1.5. Socio-Demographic Characteristics

Socio-demographic characteristics also have the potential to influence IVD international salespeople’s mental health. Several studies have shown that low income, a sales position, and risky alcohol use are risk factors for depressive tendencies [29,51]. Women have a higher lifetime prevalence of mood or anxiety disorders than men [52,53]. Educational background has been linked to some mental health challenges, such as obsessive-compulsive behaviors and anxiety symptoms [23]. Most IVD international salespeople have a medical-related educational background. Whether or not educational background, financial status, gender, alcohol use, and work experience are associated with a predisposition to psychological problems among IVD international salespeople has yet to be explored.

### 1.6. Current Study

This study aimed to determine the prevalence of depression, anxiety, and somatic symptoms among international salespeople in China’s IVD industry. We aimed to identify factors associated with the mental health of IVD international salespeople, including occupational factors, organizational factors, individual psychological factors, and socio-demographic factors.

**Figure 1:**
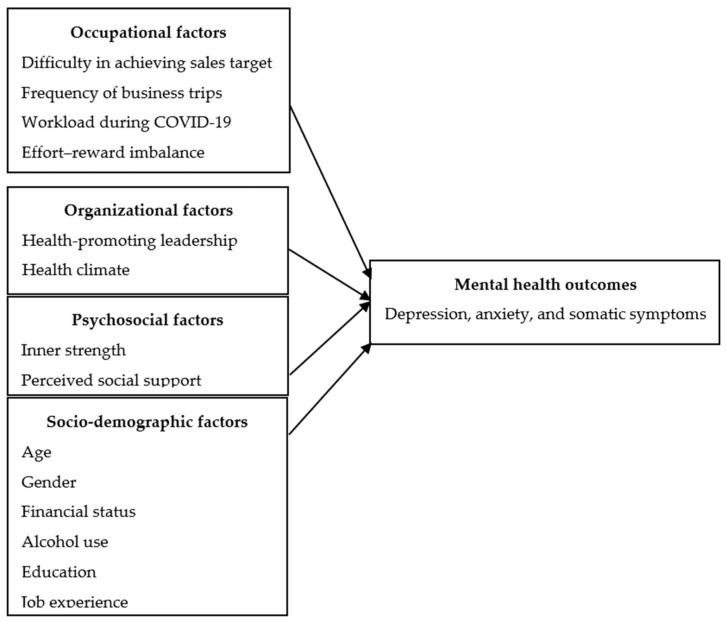
Research framework.

We hypothesized that mental health outcomes among IVD international salespeople, including depression, anxiety, and somatic symptoms, were affected by the following factors: difficulty in achieving sales targets, frequency of business trips, workload during COVID-19, effort–reward imbalance, health-promoting leadership, health climate, inner strength, perceived social support, and socio-demographic characteristics, including age, gender, financial status, alcohol use, education, and job experience (Figure 1).

## 2. Materials and Methods

### 2.1. Study Design

This was a cross-sectional study to survey the mental health of IVD international salespeople and factors affecting them in China from August 2022 to March 2023. The study investigated the factors affecting the mental health of IVD international salespeople in IVD companies officially registered in China, including local and international companies, joint ventures, sole proprietorships, state-owned, private, joint-stock, and limited liability companies. According to the 2020 China National Medical Products Administration, there were 1392 IVD manufacturers in China [54]. However, 718 IVD export companies had an international sales department, with the estimated number of IVD international salespeople being 5–10 employees [19]. It was, therefore, estimated that, in 2022, the total number of IVD international salespeople in China would be about 5000.

### 2.2. Participants

This study involved international salespeople working for China’s IVD companies, regardless of their nationality. The sampling frame included IVD international salespeople from 75 IVD companies that participated in the Medlab exhibition 2023 in Bangkok, 95 IVD companies registered with the Thai FDA, and 100 companies registered with the China IVD Association. In addition, participants were recruited using a non-probabilistic, convenient sampling method. Inclusion criteria included an international salesperson being (1) aged 18 years or older at the date of completing the questionnaire, with no maximum age limit; (2) currently employed in the international sales department of an IVD company in China; (3) responsible, primarily, for international sales work, with additional responsibilities in other departments being permitted; (4) employed for at least 3 months in an IVD company; and (5) willing to participate in the study and provide informed consent online. The exclusion criterion was that international salespeople had been on leave of absence from work for at least one month.

### 2.3. Procedure

The study was approved by the Ethics Committee of the Faculty of Pharmacy, Chiang Mai University, Thailand.

**Figure 2:**
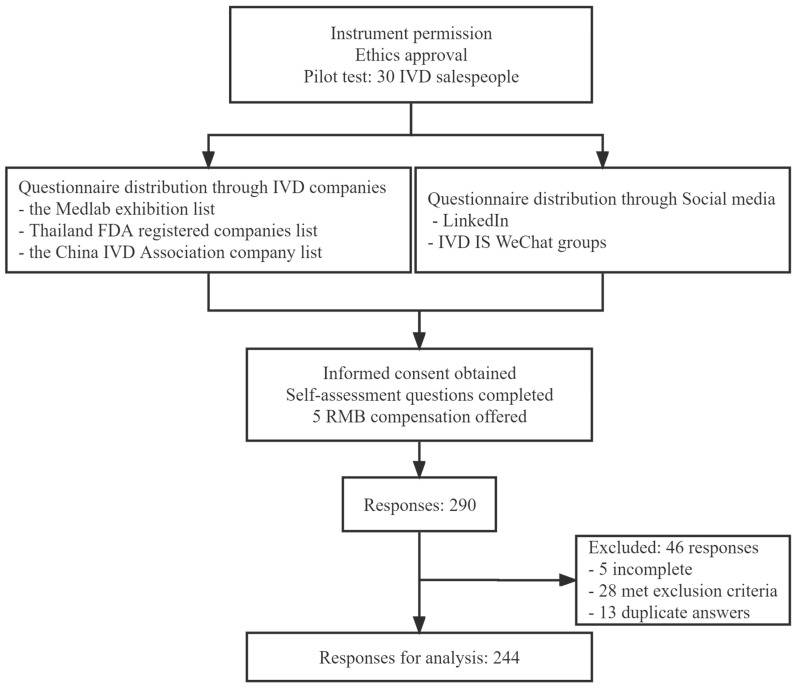
Data collection procedure.

The questionnaire was created as an online questionnaire using the Questionnaire Star platform. The researchers distributed the link and a QR code to IVD international salespeople by contacting IVD companies and posting the link on social media. The lists of IVD companies were obtained from three sources: the Medlab exhibition list, the Thailand FDA-registered companies list, and the China IVD Association company list. The researchers posted the link to the online questionnaire on social media sites such as LinkedIn and the IVD international salespeople’s WeChat groups. Participants were provided with a detailed description of the study, including a participant information sheet (PIS) and an informed consent form (ICF). Participants were informed that the questionnaire was completely voluntary and that its intended use was for research purposes only. Participants were asked to fill out self-assessment questions according to the study’s inclusion and exclusion criteria. Then the international salespeople responded to the questionnaires. Each participant received RMB 5 after completing the questionnaire as compensation for their active participation. The researchers paid the amount to the questionnaire platform, and each participant received a WeChat Red Envelope as compensation. The payments were anonymous (Figure 2).

### 2.4. Measurements

#### 2.4.1. Core Symptoms Index

The Core Symptom Index (CSI) scale was used to screen for depression, anxiety, and somatic symptoms [55]. Having been tested in long-term care institutions, the CSI scale has demonstrated reliability and predictive validity (Cronbach’s alpha 0.91). This scale includes 5 items for depression (items 2, 4, 5, 6, and 7), 4 items for anxiety (items 12, 13, 14, and 15), and 6 items for somatic symptoms (1, 3, 8, 9, 10, and 11). All responses were obtained using a 5-point Likert scale anchored from 0 (never) to 4 (almost always). The higher the CSI score, the higher the level of psychopathology indicated. The Cronbach’s alpha of the CSI Chinese version from the pilot test was 0.915. The cut-off score on the depression subscale of 9 suggests major depression, whereas the cut-off score on the anxiety subscale of 9 suggests anxiety disorder [55].

#### 2.4.2. Effort–Reward Imbalance

The effort–reward imbalance model proposes that the imbalance between high effort and low reward at work is seen as occupational stress [56]. The Chinese version of the ERI questionnaire, introduced by Li Jian [57] and later translated by Dai Junming et al. [58], demonstrated validity and reliability in a study of Chinese employees [59], with a Cronbach’s α of 0.88. The questionnaire consists of 23 Likert-scale items, including three subscales. The effort scale comprises six items to measure the level of demand in the work environment. The 11-item reward scale measures career rewards, with lower scores indicating fewer rewards. The overcommitment scale includes five items. The E–R ratio is computed by dividing the average work effort by the average reward score using the formula ER = E/(R × C), where E is effort, R is reward, and C is a correction factor equal to 0.54 (6/11). A ratio greater than 1.0 is considered occupational stress; that is, the participant reports more effort for the reward.

#### 2.4.3. Health-Promoting Leadership

Health-promoting leadership, developed by Franke, is a type of leadership that promotes organizational health-promoting management as its core task [60]. The HPL employee evaluation questionnaire includes the three dimensions of health concept, health awareness, and health behavior, mainly to evaluate the HPL from the perspective of employees. A five-point Likert-scale was used, from 1 (“strongly disagree”) to 5 (“strongly agree”). The Chinese version of HPL demonstrated high reliability and validity [35,61]. The reliability range of the HPL questionnaire is 0.84 to 0.88.

#### 2.4.4. Health Climate

The health climate scale, developed by Basen-Engquist and colleagues, is used as a measurement tool to measure the health climate factors in an organization [62]. Franke et al., suggest that a healthy work environment can influence employee attitudes and help them develop healthy living habits [33]. The scale employs the 5-point Likert scoring method and was verified by research in a Chinese enterprise as having good reliability and validity, with a reliability coefficient of 0.892 [36].

#### 2.4.5. Inner Strength-Based Inventory

The Inner Strength-Based Inventory (SBI) is an instrument to evaluate participants’ psychological characteristics based on ten perfections. The ten perfections include truthfulness, perseverance, wisdom, generosity, morality, mindfulness, patience and endurance, equanimity, determination, and loving kindness [45]. There are five multiple-choice options for each item on the questionnaire. Scores range from 10 to 50, with higher scores indicating higher levels of inner strength. The reliability test for the Chinese version of the SBI showed a good reliability coefficient of 0.86 [45].

#### 2.4.6. Multidimensional Scale of Perceived Social Support

The Multidimensional Scale of Perceived Social Support (MSPSS) is a tool used to assess how much social support a person perceives [63]. The Chinese version of the MSPSS was validated by Wang Xiangdong et al., in 1999 and is widely used in China [64,65,66]. The subscales are applicable to three different sources of social support, including family, friends, and significant others. A total of 12 items are scored using a 7-point Likert scale from “strongly disagree” to “strongly agree”, with a total score reflecting the overall degree of social support felt by the individual. The overall Cronbach’s alpha of this current sample was 0.962.

#### 2.4.7. Characteristics of Participants

Socio-demographic factors relating to IVD international sales were studied, including age, gender, marital status, financial status, education, alcohol use, and job experience in IVD (year). Occupational factors included frequency of international business trips in the previous twelve months, workload during COVID-19 compared to before COVID-19 (“significantly decreased” to “significantly increased”) [67], and difficulty in achieving sales targets (“easy to achieve”, “difficult to achieve”, and “not achievable”) [21].

A sample size of 30 IVD international salespeople was used to test the internal reliability of the instruments. The instruments proved valid with acceptable reliability (Cronbach’s α > 0.70 for all instruments).

### 2.5. Statistical Analysis

Descriptive analysis was used to describe demographic data, mental health outcomes, depression, anxiety, somatic symptom scores, and the CSI score as mean and standard deviation. Socio-demographic variables that are nominal or ordinal were summarized using numbers and percentages. *T*-tests were used to investigate the differences between groups when the variables were continuous. Chi-square tests were used to check the difference between categorical variables. Pearson’s correlation was used to identify the correlation between continuous variables. Univariate regression analysis was used for each predictor with *p* < 0.05; significant variables were then tested using multivariable regression analysis for each outcome. SPSS version 26 was used to analyze the data. For all the data, a *p*-value < 0.05 was considered significant.

## 3. Results

A total of 290 respondents participated in the study. Among them, 5 respondents provided incomplete data and 13 gave duplicate answers; the 28 respondents who did not meet the inclusion criteria were excluded. The total number of respondents used in the data analysis was 244 IVD international salespeople.

### 3.1. Socio-Demographic and Psychological Characteristics of Participants

Most of the respondents (n = 130, 53.3%) were between 25 and 34 years old; 126 men (51.6%) and 134 (54.9%) reported being in a relationship. Most respondents were educated to a bachelor’s degree level or below (79.9%), did not drink alcohol (58.8%), and had 1–3 years of IVD job experience (35.0%). Half of the respondents reported insufficient income (49.6%), and about one-fifth had been on more than three business trips in the previous twelve months (18.4). A total of 112 (45.9%) respondents indicated that the workload increased during the COVID-19 period, and 169 (69.6%) respondents indicated that sales targets were difficult to achieve or not achievable. The effort–reward imbalance was present in 78 IVD salespeople (32%). The mean and standard deviation for the CSI, HPL, HC, ISB, and PSS scores were 12.89 ± 10.68, 9.79 ± 2.63, 17.21 ± 3.96, 31.18 ± 8.08, and 54.36 ± 13.44, respectively (Table 1).

The prevalence of major depression in IVD international salespeople was 18.4%, while the prevalence of anxiety disorder was 10.2%. The prevalence of the co-occurrence of major depression and anxiety was 8.2% in this population.

### 3.2. Psychological Variables and Characteristics of Participants

As shown in Table 2, female IVD salespeople had significantly higher scores of CSI, anxiety, and somatic symptoms than males. Similarly, salespeople with insufficient income scored higher than those with sufficient income. A higher proportion of major depression was found in salespeople who were female, had insufficient income, and had less job experience. Salespeople with decreased or unchanged workload during COVID-19 showed a higher score of somatic symptoms than the ones with increased workload (*p* < 0.05).

### 3.3. Pearson’s Correlation among Psychological Variables

In Table 3, Pearson’s correlation analysis showed that the CSI–total score was positively correlated with effort–reward imbalance (r = 0.310, *p* < 0.01) and negatively correlated with inner strength (r = −0.300, *p* < 0.01) and perceived social support (r = −0.195, *p* < 0.01). Major depression was positively correlated with effort–reward imbalance (r = 0.361, *p* < 0.01) and negatively with health climate (r = −0.132, *p* < 0.05), inner strength (r = −0.306, *p* < 0.01), and perceived social support (r = −0.186, *p* < 0.01). Anxiety had a positive moderate correlation with effort–reward imbalance (r = 0.320, *p* < 0.01) and a negative correlation with inner strength (r = −0.278, *p* < 0.01) and perceived social support (r = −0.194, *p* < 0.01). Moreover, somatic symptoms were positively correlated with effort–reward imbalance (r = 0.192, *p* < 0.01) but negatively correlated with inner strength (r = −0.248, *p* < 0.01) and perceived social support (r = −0.166, *p* < 0.01). The sub-scores of the MSPSS presented similar results to the MSPSS total score for the psychological variables.

Furthermore, a significant positive correlation was observed between the core symptom index and depression (r = 0.928, *p* < 0.01), anxiety (r = 0.915, *p* < 0.01), and somatic symptoms (r = 0.926, *p* < 0.01). Depression was positively correlated with anxiety (r = 0.798, *p* < 0.01) and somatic symptoms (r = 0.763, *p* < 0.01). Similarly, anxiety was positively correlated with somatic symptoms (r = 0.778, *p* < 0.01).

Additionally, it is noteworthy that the effort–reward imbalance was negatively correlated with health-promoting leadership (r = −0.126, *p* < 0.05) and inner strength (r = −0.240, *p* < 0.01). Moreover, significant positive correlations were observed between health-promoting leadership and health climate (r = 0.743, *p* < 0.01), inner strength (r = 0.537, *p* < 0.01), and perceived social support (r = 0.530, *p* < 0.01). Health climate was positively correlated with inner strength (r = 0.534, *p* < 0.01) and perceived social support (r = 0.546, *p* < 0.01), as well as a positive correlation between inner strength and perceived social support (r = 0.481, *p* < 0.01).

### 3.4. Factors Predicting Mental Health Outcomes in IVD International Salespeople

The results in Table 4 show that gender, effort–reward imbalance, and inner strength were significant predictors of CSI total score, major depression, anxiety disorder, and somatic symptoms. Education was a predictor of somatic symptoms (*p* < 0.05) but had no effect on the CSI total score, major depression, or anxiety disorder. There was no significant association between age, marital status, financial status, and perceived social support and the CSI total score and anxiety symptoms (*p* > 0.05). Additionally, our analysis did not find any associations between age, job experience, financial status, and perceived social support for major depression in IVD international salespeople. Furthermore, financial status, workload during COVID-19, and perceived social support were not significant predictors of somatic symptoms.

## 4. Discussion

The study aimed to evaluate the mental health outcomes and potential influencing factors for international salespeople working in the IVD industry in China. As IVD products were in high demand during the COVID-19 pandemic, we hypothesized that the potential increase in work would affect the mental health of the IVD international salespeople. While prior studies on mental health outcomes in employees in industries have mainly focused on the effect of the individual’s sociodemographic factors such as income, education, and gender on mental health, this study extended the investigation into the effect of occupational factors, such as workload and effort–reward imbalance, organizational factors such as health climate and health-promoting leadership, and mental health-promoting factors, such as inner strengths and perceived social support, on mental health outcomes.

This study found that the prevalence of major depression and anxiety disorders was 18.4% and 10.2%, respectively, in international salespeople in China’s IVD industry. The comorbidity rate for severe depression and anxiety in this population was 8.2%. Current research shows a lower prevalence of major depression and anxiety in IVD international salespeople compared with a study on the prevalence of depression and anxiety among the general population during the COVID-19 lockdown in Shanghai in 2022 (26.1% and 20.1%, respectively [68]). The lower prevalence of depression and anxiety among IVD salespeople compared with the rates during the COVID-19 pandemic might be explained by different reasons. The first half of the survey period covered the period when strict lockdown measures were implemented in China. Several studies have documented increased mental health burdens associated with strict lockdown measures [68,69,70]. However, in the latter half of the data collection period since 8 January 2023, China downgraded its virus control measures from Category A to Category B [71]. This adjustment might reduce the impact of COVID-19 on the mental health of IVD salespeople. However, the prevalence of major depression and anxiety in IVD international salespeople was greater compared with the pre-covid prevalence in adults in China, which was 8.6% and 6.0%, respectively [72]. However, such significant disparities in the prevalence of major depression and anxiety from this study post-COVID compared with during and post-COVID-19 pandemic might be attributed to the timing of the survey, the COVID-19 prevention measures in China, the context of the IVD industry, and the use of different instruments to screen depression and anxiety [73]. This study used the CSI scale to assess major depression in IVD salespeople in China, while the study of major depression in salespeople in Pakistan [21] used the PHQ-8 scale [73].

Regarding somatic symptoms, the participants reported low levels of symptoms compared with other professions, e.g., medical staff [74]. In comparison of the mean scores of somatic symptoms of Thai clinical elder adults and the international salespeople using the CSI scale, the clinical samples had a significantly higher score than the IVD salespeople in China [55]. Notably, somatic symptoms are culturally sensitive [75,76], and many emotional expressions in the Chinese language fail to make a clear distinction between physical symptoms and psychological distress [77]. In addition, somatic symptoms are usually related to other symptoms, i.e., depression and anxiety [78,79].

The current study discovered that some socio-demographic variables were associated with CSI symptoms. Associations between gender and CSI total score, depression, anxiety, and somatic symptoms were found. Females indicated a higher prevalence of depression, anxiety, and somatic symptoms than males. Higher education levels were associated with a lower score of somatic symptoms. Mental health disparities between women and men have received attention in many fields related to mental health. The literature consistently reports that anxiety and depression disorders are more prevalent in women than in men [80,81]. This difference occurs not only in adults but also in children and adolescents [82]. Women are more likely than men to report somatic symptoms, including appetite and weight disturbances and fatigue [83,84,85]. The gender differences in anxiety disorders are affected by social, psychological, and biological factors [81]. Previous studies have indicated that higher levels of education are associated with a lower risk of somatic symptoms and provide protective effects against depression, Alzheimer’s disease, and hypertension [86,87,88,89]. The correlation between increased education levels and health outcomes has been validated. Compared with other related studies, no effects of age, income, alcohol use, workloads during COVID-19, or job experience were observed. The observed results might be explained by limited statistical power or the involvement of other intervening factors that require a more comprehensive investigation.

Our study demonstrated a positive correlation between ERI, an occupational factor, and depression, anxiety, and somatic symptoms. The magnitude of occupational stress from ERI in IVD international salespeople was lower compared with that in workers in Internet companies in China [29]. In addition to attractive salary packages, IVD companies offer insurance benefits, annual health assessments, and international career development opportunities. Moreover, considering that IVD international salespeople are often required to work late hours to accommodate time zone differences, companies adopt a flexible working system where employees have more flexible working hours and locations [90]. This improves employee efficiency and job satisfaction while reducing employee stress [91,92]. Additionally, ERI was identified as a significant predictor for both the high CSI score and more severe depression, anxiety, and somatic symptoms. The higher the level of ERI, indicating occupational stress, the worse the individual’s mental state [93], which has been confirmed in studies across different industries and organizations. ERI as occupational stress was found to be the primary cause of depression in a Canadian occupational population study [94], and a significant association between ERI and depressive symptoms was observed among Turkish university staff [95]. A study on the relationship between occupational stress and depression tendencies among employees of Internet companies in China confirmed that ERI-type occupational stress increases the risk of depression [29].

It has been demonstrated that the health climate was negatively correlated with participants’ depression. Consistent with previous research, workplace health climate was negatively correlated with work pressure and fatigue [96], and a team’s health climate was positively correlated with the general and mental health of employees [97]. In other words, multiple studies support that a health climate is a contextual resource, and a positive and supportive team-health climate was more beneficial to employees’ mental health-related outcomes [39,98]. Interestingly, this study did not find a statistically significant association between HPL and depression, anxiety, or somatic symptoms. This might be due to the fact that the respondents in this study included both leaders and employees of IVD international sales without distinguishing between their job positions, the perspective on HPL might vary widely between the two. A study conducted in Germany on the relationship between health-oriented leadership and mental health showed that only the subjective assessment of employees could significantly predict their mental health [34]. It is important to note that these variables are not the actual representative organizational factor but the ‘perception’ of employees about health-promoting leadership. Moreover, such perceptions may differ greatly from the self-perceptions of leaders. Future research should explore the complex relationship between multisource assessments of HPL and employee mental health, as well as other objective assessments of organizational factors.

We found a significant negative correlation between inner strength and CSI score, depression, anxiety, and somatic symptoms, which was in line with previous research [43,99,100]. It is important to note that while the inner strength measurement scale used in the current study was based on ten perfections, e.g., mindfulness and perseverance [45], the results are consistent with another theory of inner strength, e.g., connectedness, firmness, flexibility, and creativity, proposed by Lundman [101]. Based on the ten-perfection concept of inner strength, our study highlighted that inner strength is a key predictor of CSI score, depression, anxiety, and somatization. Given these findings, international salespeople of IVDs with greater inner strength are more likely to achieve the highest levels of mental health.

The current study confirmed a negative correlation between perceived social support and CSI total scores, depression, anxiety, and somatic symptoms. We also found that IVD international salespeople in China perceived a high level of social support, accounting for 33.2%. Despite the high perceived social support among IVD salespeople, this study did not find any association between perceived social support and mental health outcomes. Several patient-focused studies found that high levels of social support could reduce patients’ levels of depression and anxiety [102,103]. The mechanism explaining this association is alexithymia, that is, feeling isolated and unsupported when facing problems and having difficulty expressing one’s emotions, which leads to increased psychological stress [104].

Combined with the findings discussed above, our study advocates that improving the balance between effort and reward and inner strength would have a positive effect on the mental health of IVD international salespeople [46,105]. It is recommended that the IVD industry employer design a salary system that reflects a good balance of effort and reward, as well as training and activities to promote inner strengths for the employees. In addition, employers should establish a mental health assessment mechanism for IVD international salespeople, particularly focusing on high-risk groups with poor mental health, such as female employees. Regular mental health surveys help facilitate the understanding of the current mental health status of IVD international salespeople.

### Strengths and Limitations

This study constituted the first study exploring risk factors in occupation and organization among international salespeople in IVD industries in China affected by COVID-19 on the supply side. The effects of inner strength on promoting mental health outcomes in IVD international salespeople found in this study confirm the importance of positive psychology not only for women and patient groups but also for industry employees. This study found a significant relationship between effort–reward imbalance and employees’ mental health, suggesting that the IVD industries might put more effort into creating human resource management systems that promote the balance of effort and reward systems. The study’s sample size is sufficient for the statistical analyses.

Although the study’s results are informative, there are some limitations. The participants we invited were not categorized based on job titles. In future studies, it is important to differentiate between employees and leaders in terms of their positions to better explain health-promoting leadership. Other variables that might affect job stress and cause psychological problems in IVD international salespeople, such as organizational size, number of employees, and personality traits, were not examined.

## 5. Conclusions

The prevalence of depression and anxiety in China’s IVD international salespeople post-COVID-19 was 18.4% and 10.2%, respectively, and was considered low compared with general populations during COVID-19 but higher than those before the pandemic [68,72]. Female employees significantly presented more depression, anxiety, and somatic symptoms than male employees. Higher education levels were associated with a lower risk of somatic symptoms. This study found that employees with an effort–reward imbalance were more likely to have depression, anxiety, and somatic symptoms. Inner strength was a significant factor in promoting mental health among IVD international salespeople in China. Organizational factors, health-promoting leadership and health climate, age, financial status, alcohol use, job experience, business trips, workload during COVID-19, and difficulty in achieving sales targets were not found to be associated with mental health outcomes in IVD international salespeople.

## Figures and Tables

**Table 1:** Participant characteristics by percentage, mean, and SD (n = 244).

Variables	Mean (SD) or n (%)
Age, n (%)	
18–24	34 (13.9)
25–34	130 (53.3)
35–44	67 (27.5)
44–54	11 (4.5)
54 and order	2 (0.8)
Gender, n (%)	
Male	126 (51.6)
Female	118 (48.4)
Financial status, n (%)	
Not enough income or incurring debt	19 (7.8)
Barely sufficient income, adequate income without debt	102 (41.8)
Enough income without savings	56 (23.0)
Enough income with some savings	67 (27.4)
Alcohol use, n (%)	
Yes	100 (41.2)
No	143 (58.8)
Educational, n (%)	
Bachelor’s degree or below	195 (79.9)
Master’s degree or above	49 (20.1)
Job experience, n (%)	
Less than a year	52 (21.4)
1–3 years	85 (35.0)
4–6 years	57 (23.4)
More than 6 years	49 (20.2)
Marital status, n (%)	
Single	108 (44.3)
Married/living together/cohabiting	134 (54.9)
Divorced/separated	2 (0.8)
Occupational factors	
Sales target, n (%)	
Easily achievable	74 (30.4)
Difficult to achieve	152 (62.6)
Not achievable	17 (7.0)
Frequency of business trip, n (%)	
0 trips/year	98 (40.2)
1–3 trips/year	101 (41.4)
>3 trips/year	45 (18.4)
Workload during COVID-19, n (%)	
Significantly decreased	55 (22.5)
Decreased	40 (16.4)
Not changed	37 (15.2)
Increased	74 (30.3)
Significantly increased	38 (15.6)
Effort–reward imbalance (>1 imbalance), n (%)	78 (32.0)
Organizational factors	
Health-promoting leadership (range 0–15)	9.79 (2.63)
Health climate (range 0–25)	17.21 (3.96)
Psychological factors	
Inner strength (range 10–50)	31.18 (8.08)
Perceived social support–total score (range 12–84)	54.36 (13.44)
Perceived social support from significant others (mean scores range 1–7)	4.43 (1.20)
Perceived social support from family members (mean scores range 1–7)	4.48 (1.16)
Perceived social support from friends (mean scores range 1–7)	4.57 (1.27)
Mental health outcomes, n (%) and mean (SD)	
CSI total score (range 0–60)	12.89 (10.68)
CSI-depression score (range 0–18)	4.69 (4.11)
CSI-anxiety score (range 0–13)	3.81 (3.00)
CSI-somatization (somatic symptoms) (range 0–17)	4.38 (4.32)
Major depression (CSI depression score ≥ 9), n (%)	45 (18.4)
Anxiety disorder (CSI anxiety score ≥ 9), n (%)	25 (10.2)

**Table 2:** Percentage, mean, and SD of psychological variables in participant characteristics (n = 244).

Variables	n (%)	CSI Total Score		Anxiety Score		Somatic Score		Major Depression		
Age		Mean ± SD	*p*-Value	Mean ± SD	*p*-Value	Mean ± SD	*p*-Value	Non-MD N (%)	MD N (%)	*p*-Value
35 or older	80 (32.8) 164 (67.2)	10.36 ± 9.88 14.13 ± 10.87	0.009	2.98 ± 2.82 4.23 ± 3.20	0.003	3.91 ± 4.23 4.61 ± 4.36	0.238	71 (35.7) 128 (64.3)	9 (20.0) 36 (80.0)	0.053
18–34 years										
Gender										
Male	126 (51.6) 118 (48.4)	10.05 ± 8.54 15.93 ± 11.88	<0.001	3.17 ± 2.49 4.51 ± 3.57	<0.001	3.34 ± 3.61 5.49 ± 4.74	<0.001	116 (58.3) 83 (41.7)	10 (22.2) 35 (77.8)	<0.001
Female										
Financial status										
Sufficient income	123 (50.4) 121 (49.6)	11.01 ± 10.11 14.80 ± 10.95	0.005	3.37 ± 2.98 4.26 ± 3.23	0.026	3.77 ± 4.01 5.00 ± 4.55	0.026	109 (54.8) 90 (45.2)	14 (31.1) 31 (68.9)	0.005
Insufficient income										
Alcohol use										
No Yes	143 (58.8) 100 (41.2)	12.20 ± 10.19 13.88 ± 11.39	0.230	3.66 ± 2.95 4.06 ± 3.39	0.326	4.10 ± 4.21 4.76 ± 4.49	0.247	120 (60.6) 78 (39.4)	23 (51.1) 22 (48.9)	0.246
Education										
Bachelor’s degree or below	195 (79.9) 49 (20.1)	13.27 ± 10.84 11.41 ± 10.02	0.277	3.85 ± 3.11 3.67 ± 3.23	0.723	4.66 ± 4.41 3.27 ± 3.78	0.043	158 (79.4) 41 (20.6)	37 (82.2) 8 (17.8)	0.837
Master’s degree or above										
Job experience										
More than 1 year	191 (78.6) 52 (21.4)	12.25 ± 10.26 15.13 ±12.02	0.085	3.67 ± 3.05 4.33 ± 3.41	0.181	4.19 ± 4.19 5.08 ± 4.79	0.190	165 (82.9) 34 (17.1)	26 (59.1) 18 (40.9)	<0.001
Less than a year										
Marital status										
In relationship	134 (54.9) 110 (45.1)	11.31 ± 10.14 14.82 ± 11.05	0.010	3.36 ± 2.98 4.37 ± 3.23	0.012	4.07 ± 4.34 4.76 ± 4.29	0.211	113 (56.8) 86 (43.2)	21 (46.7) 24 (53.3)	0.247
Single										
Frequency of business trips										
0 trips/year	199 (81.6) 45 (18.4)	13.48 ± 10.81 10.29 ± 9.82	0.070	3.98 ± 3.14 3.09 ± 3.03	0.085	4.48 ± 4.35 3.96 ± 4.23	0.466	158 (79.4) 41 (20.6)	41 (91.1) 4 (8.9)	0.087
>1 trips/year										
Workload during COVID-19										
Decreased or not changed	132 (54.1) 112 (45.9)	13.27 ± 10.24 12.45 ± 11.21	0.055	3.80 ± 3.09 3.83 ± 3.19	0.946	4.89 ± 4.10 3.78 ± 4.50	0.044	105 (52.8) 94 (47.2)	27 (60.0) 18 (40.0)	0.411
Increased										
Sales target										
Easy to achieve	74 (30.5) 169 (69.5)	12.28 ± 10.49 13.12 ± 10.81	0.574	3.36± 3.06 4.00 ± 3.16	0.143	4.89 ± 4.31 4.15 ± 4.33	0.222	63 (31.7) 136 (68.3)	11 (25.0) 33 (75.0)	0.470
Difficult or not achievable										

Non-MD: Non-Major Depression. MD: Major Depression. In relationship: Married/living together/cohabiting. Single: Single/Divorced/Separated. Bachelor’s degree or below: Secondary school, high school, college or undergraduate. Sufficient income: Enough without saving/Enough with some saving. Insufficient income: Not enough, incurring debt/Barely sufficient, adequate (without debt).

**Table 3:** Correlation coefficients among psychological variables.

Variable	CSI	Depression	Anxiety	Somatic	ERI	HPL	HC	SBI	MSPSS Total	MSPSS— Family	MSPSS— Friends	MSPSS—SO
CSI	1											
Depression	0.928 **	1										
Anxiety	0.915 **	0.798 **	1									
Somatic symptom	0.926 **	0.763 **	0.778 **	1								
ERI	0.310 **	0.361 **	0.320 **	0.192 **	1							
HPL	−0.092	−0.113	−0.085	−0.059	−0.126 *	1						
HC	−0.115	−0.132 *	−0.053	−0.120	−0.096	0.743 **	1					
SBI	−0.300 **	−0.306 **	−0.278 **	−0.248 **	−0.240 **	0.537 **	0.534 **	1				
MSPSS—Total	−0.195 **	−0.186 **	−0.194 **	−0.166 **	−0.012	0.550 **	0.546 **	0.481 **	1			
MSPSS—family members	−0.228 **	−0.237 **	−0.223 **	−0.176 **	−0.044	0.529 **	0.534 **	0.411 **	0.896 **	1		
MSPSS—friends	−0.147 *	−0.128 *	−0.165 **	−0.121	0.014	0.490 **	0.449 **	0.421 **	0.932 **	0.816 **	1	
MSPSS—significant others	−0.163 *	−0.153 *	−0.164 *	−0.139 *	−0.035	0.535 **	0.549 **	0.469 **	0.921 **	0.726 **	0.781 **	1

* *p* < 0.05, ** *p* < 0.01 (CSI—core symptom index; ERI—effort reward imbalance; HPL—health promoting leadership; HC—health climate; SBI—inner Strength-based inventory; MSPSS—Multidimensional scale of perceived social support; SO—significant others).

**Table 4:** Multivariable regression analysis of factors predicting CSI total score, major depression, anxiety, and somatic symptoms among IVD international salespeople (n = 244).

Variable	Predictor	B	SE	β	*p*	95% LL-CI	95% UL-CI
CSI total score ***	Age	1.502	1.497	0.066	0.317	−1.447	4.451
	Gender	3.898	1.285	0.183	0.003	1.366	6.430
	Marital status	0.021	1.416	0.001	0.988	−2.768	2.809
	Financial status	−0.233	1.328	−0.011	0.861	−2.849	2.384
	ERI–score	7.132	1.352	0.312	0.000	4.468	9.795
	SBI–score	−0.217	0.091	−0.164	0.018	−0.395	−0.038
	MSPSS–total score	−0.091	0.053	−0.114	0.086	−0.195	0.013
Major depression **	Age	−0.629	0.501	1.875	0.209	0.703	5.003
	Gender	−1.399	0.443	4.052	0.002	1.702	9.647
	Job experience	−0.725	0.441	2.065	0.100	0.870	4.900
	Financial status	−0.083	0.432	1.086	0.848	0.465	2.535
	ERI–score	−1.988	0.434	7.303	0.000	3.119	17.103
	SBI–score	−0.083	0.032	0.920	0.009	0.865	0.979
	MSPSS–total score	−0.028	0.016	0.973	0.087	0.942	1.004
Anxiety score ***	Age	0.704	0.450	0.106	0.119	−0.181	1.590
	Gender	0.813	0.386	0.130	0.036	0.053	1.574
	Marital status	−0.011	0.425	−0.002	0.980	−0.848	0.827
	Financial status	−0.236	0.399	−0.038	0.554	−1.022	0.550
	ERI–score	1.958	0.406	0.292	0.000	1.158	2.758
	SBI–score	−0.059	0.027	−0.152	0.031	−0.113	−0.005
	MSPSS–total score	−0.029	0.016	−0.124	0.069	−0.060	0.002
Somatic score ***	Gender	1.508	0.536	0.175	0.005	0.451	2.565
	Education	−1.301	0.647	−0.121	0.046	−2.576	−0.025
	Financial status	−0.243	0.551	−0.028	0.659	−1.328	0.842
	Workload during COVID-19	−1.056	0.542	−0.122	0.052	−2.123	0.011
	ERI–score	2.454	0.570	0.265	0.000	1.332	3.577
	SBI–score	−0.078	0.038	−0.146	0.039	−0.153	−0.004
	MSPSS–total score	−0.023	0.022	−0.073	0.289	−0.067	0.020

** Multivariable logistic regression analysis, α < 0.05; *** Multivariable linear regression analysis, α < 0.05; B—Unstandardized coefficient; SE—Standard Error; β—Standardized coefficient; LL-CI—lower limit confidence interval; UL-CI—upper limit confidence interval.

## Data Availability

The data presented in this study are available on request from the corresponding author. The data are not publicly available due to ethical restriction.
